# Description of patterns of ear and tail lesions during the grower-finisher period in a commercial pig farm

**DOI:** 10.1186/s40813-024-00374-w

**Published:** 2024-06-27

**Authors:** Nienke van Staaveren, Joana Pessoa, Laura Ann Boyle, Julia Adriana Calderón Díaz

**Affiliations:** 1 Department of Population Health Sciences, Faculty of Veterinary Medicine, Utrecht University, Utrecht, The Netherlands; 2 Department of Animal Biosciences, Ontario Agricultural College, University of Guelph, Guelph, ON Canada; 3 Technical University of Denmark, National Food Institute, Kemitorvet 204, Kongens Lyngby, 2800 Denmark; 4 Pig Development Department, Teagasc Animal and Grassland Research and Innovation Centre, Moorepark, Co. Cork, Fermoy, Ireland; 5 PIC Europe, Sant Cugat del Valles, C/Pau Vila, 22 2o piso, Barcelona, 08174 Spain

**Keywords:** Production diseases, Swine, Welfare, Ear necrosis, Tail biting

## Abstract

**Background:**

Ear and tail lesions are prevalent indicators of impaired welfare observed in pig production with different multifactorial causes. Understanding the progression of ear and tail lesions over time is important to implement preventative strategies on commercial pig farms. Therefore, this case study aimed to provide a detailed account of patterns of ear and tail lesions in pigs on a single commercial farm during the grower-finisher period.

**Case presentation:**

A total of 1,676 12-week old pigs (*n* = 773 females and *n* = 903 males, all tail docked) were followed from arrival to the grower facilities until transferred to the finisher stage on a commercial pig farm in Ireland. Pigs were individually weighed and inspected for the severity of fresh ear and tail lesions (score 0–4) on transfer to the first grower (24.9 ± 5.33 kg, 12 weeks of age, *n* = 1,676 pigs), second grower (33.3 ± 7.04 kg, 14 weeks of age, *n* = 1,641 pigs), and finisher stage (60.2 ± 7.74 kg, 18 weeks of age, *n* = 1,626 pigs). Due to the low number of pigs with high scores, ear lesions were classified as no (score 0), mild (score 1), moderate (score 2) and severe (score ≥ 3) and tail lesions were classified as no (score 0), mild (score 1), and moderate-to-severe (score ≥ 2). Ear lesions were more prevalent than tail lesions at each inspection. There were approx. 19% of pigs with ear lesions at all three inspections but no pigs presented with tail lesions at all three inspections. When considering the specific severity categories, we observed 32 different ear lesion score combinations and 15 different tail lesion score combinations across the three inspections.

**Conclusion:**

The high number of observed patterns of ear and tail lesions suggest large individual variability in lesion progression. Ear lesions were more of an issue than tail lesions and little is known about this health and welfare problem indicating that further research into causes and management strategies is needed.

## Background

Ear and tail lesions are prevalent welfare outcomes observed in pig production [1–3]. In the case of tail lesions, it is well documented that they are caused by tail biting, while a similar relationship between ear biting and ear lesions is more tentative and received only little attention in the past couple of years [4, 5]. Many studies investigating tail or ear lesions are based on cross-sectional observations of pigs in different production stages [1–3], while fewer studies followed pigs over time [6–9]. However, longitudinal studies provide valuable insights into the development of health and welfare conditions over time [6, 8] or the classification of farms by their health status [10]. For example, Diana et al. [6] observed that ear lesions were less likely in older pigs (9–16 weeks of age), while tail lesions were more likely in pigs of 24 weeks of age compared to younger pigs (7 weeks of age). Understanding when lesions occur during a pig’s productive life is necessary to target interventions and optimize management strategies to prevent/reduce the occurrence of ear and tail lesions. A further complication is that ear and tail lesions, which can be recorded on detailed scoring scales [11, 12], are often presented as a binary trait (presence/absence) for practical/statistical reasons. This loss in granularity means that there are few descriptions of how the severity of ear and tail lesions progresses (e.g., when do lesions deteriorate from mild to severe). Finally, there are few reports on the co-occurrence of ear and tail lesions even though this could potentially confound specific research questions [13]. Co-occurrence may also be of value in determining appropriate management strategies to address the cause of lesions [14], especially considering that ear and tail lesions are multifactorial but may share similar risk factors [3, 6, 15]. Therefore, the aim of this case study is to provide an account of patterns of ear and tail lesions in pigs on a single commercial farm in Ireland during the grower-finisher period.

## Case presentation

This was an observational study conducted on a grower-finisher pig farm in Ireland between July and November 2018. The farm was positive for *Mycoplasma hyopneumoniae* (Mhyo), *Actinobacillus pleuropneumoniae* (APP), porcine reproductive and respiratory syndrome virus (PRRSv) and Influenza A virus (IAv) and vaccinated for Mhyo, PRRSv, and IAv. During the study, a total of 1,676 12-week old pigs (*n* = 773 females and *n* = 903 males) were followed from arrival to the grower facility until transfer to the finisher stage. Pigs were managed as per routine farm practice; all pigs were tail docked and teeth clipped at birth and males were not castrated. On arrival at the grower-finisher farm each pig was individually ear tagged and housed in mixed sex groups of approx. 36 pigs in concrete pens with fully slatted floors. Pigs remained in the same groups throughout the production stages, i.e., they were not remixed at any stage. Hard-plastic balls were provided to pigs in each pen. Feed was delivered three times per day (Hydromix wet feeding system, Big Dutchman, IDS, Portlaoise, Co. Laois, Ireland) and water was provided *ad libitum*. Environmental conditions were controlled through an automatic temperature-based control system with roof-mounted exhaust fans (Big Dutchman) from 12 to 18 weeks and natural ventilation thereafter.

All pigs were inspected for ear and tail lesions by one assessor and individually weighed on arrival at the farm in the first grower stage [24.9 ± 5.33 kg of body weight (BW); 12 weeks of age], after two weeks when transferred to the second grower stage (33.3 ± 7.04 kg BW; 14 weeks of age), and after four weeks when transferred to the finisher stage (60.2 ± 7.74 kg BW; 18 weeks of age). ***Ear lesions*** were scored using a modified version of the lesion scoring system described by Diana et al. [11]. In brief, scoring was done on a 5-point scale where 0 = no lesion; 1 = mild lesions (superficial bites but no blood); 2 = moderate lesions (evidence of bites/teeth marks with fresh blood and/or infection); 3 = severe (partial total loss of the ear); and 4 = very severe (total loss of the ear). ***Tail lesions*** were scored as per Harley et al. [12] on a 5-point scale where 0 = no evidence of tail biting; 1 = evidence of chewing or puncture wounds, but no evidence of swelling; 2 = evidence of chewing with swelling and signs of possible infection; 3 = partial loss of the tail and 4 = total loss of the tail. As pigs’ tails were docked, it should be noted that scoring of partial or total loss of the tail was relative to the docked length and did not reflect the loss of tissue due to tail docking itself. Due to the low number of pigs with total loss of ears (score 4), ear lesion score 3 and score 4 were combined and classified as *severe*. For similar reasons, tail lesion score ≥ 2 were combined and classified as *moderate-to-severe.* Changes in the presence (score > 0) of ear and tail lesions across the different stages as well as changes in severity scores were assessed for each pig and different lesion patterns were identified. Furthermore, recurrent event survival analysis via Cox regression was performed in R v4.31.1 [16] to investigate the association between pig body weight and sex and the risk of having an ear or tail lesion. In a recurrent events analysis, an individual is at risk for the same event throughout the follow-up period, regardless of whether an event has occurred or not.

## Results

Twelve pigs (0.7%) died during the study period. Furthermore, a total of 38 (2.3%) pigs were removed from the study based on the farm staff decisions. Reasons for removal included lameness, clinical signs of respiratory issues, or other illnesses (e.g., meningitis), failure to thrive (e.g., small/thin pigs), or other production diseases such as hernias or prolapses. Twenty-nine pigs (76.3% out of 38) were removed during the first grower stage and 9 pigs (23.7%) were removed during the second grower stage. Hence, descriptions of lesion prevalence and patterns were based on a total of 1,676 pigs on arrival at the farm (i.e., at transfer to the first grower stage), 1,641 pigs on transfer to the second grower stage, and 1,626 pigs on transfer to the finisher stage.

The number of female and male pigs with different ear and tail lesion scores on arrival or transfer to each production stages are presented in Table 1. In general, a similar proportion of female and male pigs were affected by ear or tail lesions of differing severity at each inspection (Table 1). Pigs with ear lesions (score ≥ 1) were numerically lighter than pigs without ear lesions (score 0) in the different production stages (2.4 kg lighter on transfer to the first grower stage, 4.8 kg lighter on transfer to the second grower stage, and 3.4 kg lighter on transfer to finisher stage). This trend was less clear for pigs with tail lesions (score ≥ 1) compared to pigs without tail lesions.

**Table 1:** Number and percentage of female and male pigs with ear and tail lesions according to severity scores upon transfer to the first grower (12 weeks old), second grower (14 weeks old), and finisher (18 weeks old) stage. Average body weight (BW, in kg) within each lesion score category is also reported

		First grower						Second grower						Finisher			
	Female			Male			Female			Male			Female			Male	
	n (%)	BW, kg		n (%)	BW, kg		n (%)	BW, kg		n (%)	BW, kg		n (%)	BW, kg		n (%)	BW, kg
*Ear* *lesions* ^*1*^																	
0	448 (26.7)	26.2 ± 5.31		569 (34.0)	25.5 ± 5.37		519 (31.6)	34.6 ± 6.85		652 (39.8)	33.8 ± 6.94		479 (29.5)	61.3 ± 7.62		600 (36.9)	61.2 ± 7.99
1	113 (6.7)	23.9 ± 5.64		122 (7.3)	23.9 ± 5.41		29 (1.8)	26.5 ± 6.83		24 (1.5)	27.8 ± 5.82		0 (0)	-		0 (0)	-
2	72 (4.3)	23.2 ± 3.86		75 (4.5)	22.9 ± 4.45		8 (0.5)	28.6 ± 4.96		16 (1.0)	29.6 ± 4.17		0 (0)	-		1 (0.1)	70
≥ 3	140 (8.4)	23.5 ± 5.04		136 (8.1)	22.7 ± 4.55		199 (12.1)	31.8 ± 7.37		193 (11.8)	31.5 ± 6.42		271 (16.7)	57.8 ± 7.34		275 (16.9)	57.8 ± 7.95
*Tail lesions* ^*2*^																	
0	755 (45.0)	25.1 ± 5.34		878 (52.4)	24.6 ± 5.33		676 (41.2)	33.7 ± 7.25		760 (46.3)	33.2 ± 6.96		661 (40.7)	60.1 ± 7.72		774 (47.6)	60.1 ± 8.10
1	17 (1.0)	25.9 ± 4.83		23 (1.4)	25.3 ± 4.69		74 (4.5)	32.4 ± 6.74		104 (6.3)	32.4 ± 6.47		72 (4.4)	59.9 ± 7.81		67 (4.1)	60.9 ± 8.45
≥ 2	1 (0.1)	24.5		2 (0.1)	26.5 ± 5.66		6 (0.4)	26.2 ± 5.47		21 (1.3)	32.3 ± 6.67		17 (1.0)	58.9 ± 6.45		35 (2.2)	58.2 ± 8.08

^1^ 0 = no ear lesion; 1 = mild ear lesions (superficial bites but no blood); 2 = moderate lesions (evidence of bites/teeth marks with fresh blood and/or infection); ≥3 = severe (partial or total loss of the ear)

^2^ 0 = no tail lesions (no evidence of tail biting); 1 = mild tail lesions (evidence of chewing or puncture wounds, but no evidence of swelling); ≥2 = moderate-to-severe tail lesions (evidence of chewing with swelling and signs of possible infection, or partial/total loss of the tail)

### Patterns of ear and tail lesions across production stages

**Fig. 1:**
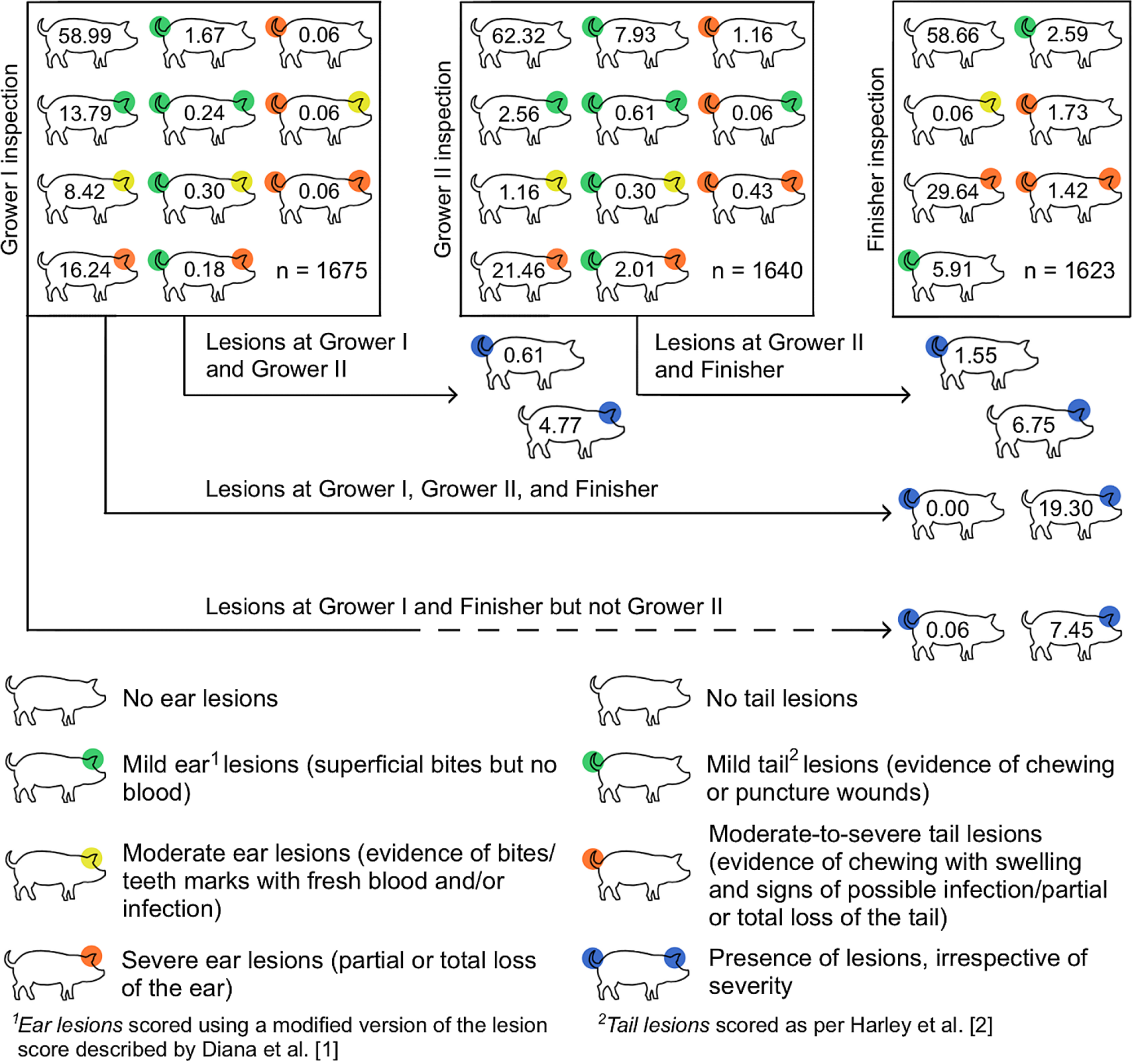
Percentage of pigs with ear and tail lesions on transfer to the first grower (Grower I inspection), second grower (Grower II inspection) and finisher (Finisher inspection) stages in a longitudinal study in grower-finisher pigs on a commercial Irish pig farm. Pigs within square boxes represent the percentage of pigs with different lesions according to severity indicated by colours. Arrows indicate the percentage of pigs with lesions (irrespective of severity) that were observed with lesions at multiple inspections

**Fig. 2:**
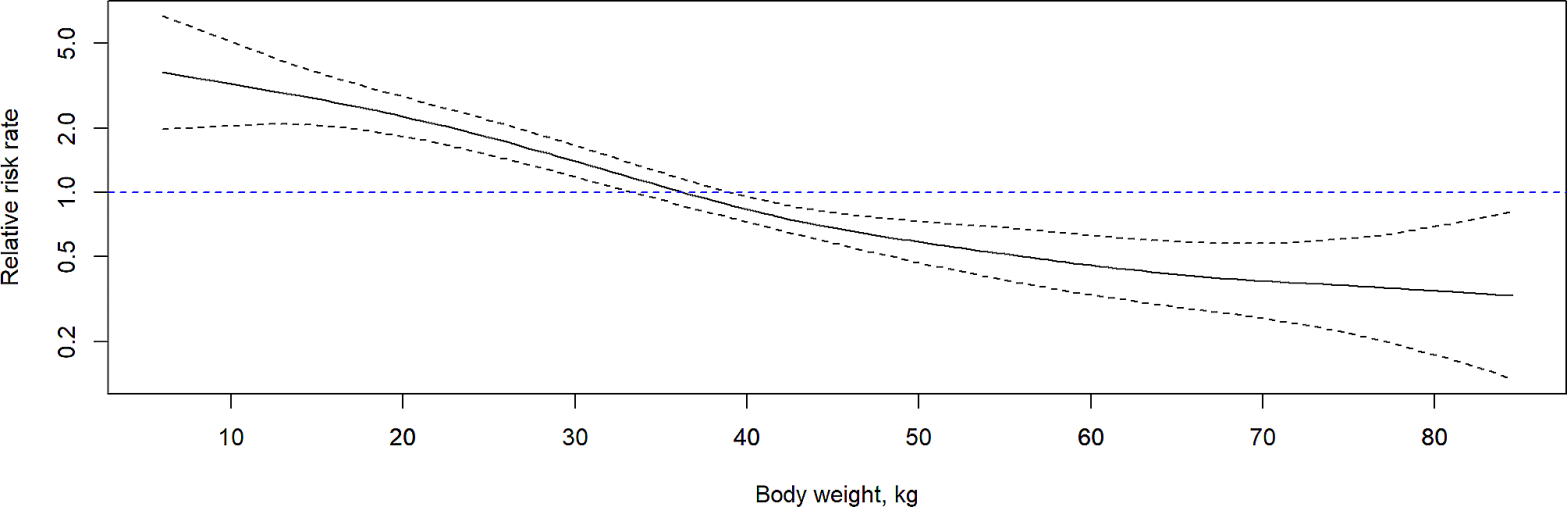
Association between pig body weight and the relative risk rate of developing an ear lesion across production stages using recurrent event survival analysis implemented in R v4.3.1. The solid black line indicates the relative risk rate with the dashed black lines indicating the 95% confidence interval

A low percentage of pigs were affected by both ear and tail lesions on transfer to the first grower (0.8%), second grower (3.4%) and finisher (4.0%) stages (Fig. 1). In total, 39.3% of first grower, 28.6% of second grower and 33.6% of finisher pigs presented ear lesions (Fig. 1). On arrival to the first grower stage, a relatively large proportion of the ear lesions were mild (14.0%) to moderate (8.8%) though 16.5% of pigs presented with severe ear lesions. As pigs progressed through the production stages, the percentage of pigs with severe ear lesions increased, while pigs with mild or moderate lesions were rarely observed. Irrespective of ear lesion severity, 19.3% of pigs presented with ear lesions at all three inspections, with lower percentages of pigs presenting ear lesions between two production stages. In particular, 7.4% of pigs with ear lesions on arrival to the first grower stage also presented with ear lesions on transfer to the finisher stage but not on transfer to the second grower stage. This was followed by 6.7% of pigs that had ear lesions on both transfer to the second grower and finisher stage, and finally, 4.8% of pigs that had ear lesions on both transfer to first grower and the second grower stage. Additionally, 11.5%, 1.7% and 4.8% of pigs only presented ear lesions on transfer to the first grower, second grower and finisher stage, respectively. Incidence of ear lesions on arrival to the second grower stage was 5.7%, while incidence of ear lesions on arrival to the finisher stage was 10%. Results from the recurrent event survival analysis showed a non-linear inverse relationship between body weight and risk of developing an ear lesion (*P* < 0.001, Fig. 2). No association was observed between sex and relative risk of developing an ear lesion (*P* > 0.05).

In terms of tail lesions, 2.6% of pigs at the first grower inspection, 12.5% of pigs at the second grower inspection and 11.7% of pigs at the finisher stage inspection were affected by lesions irrespective of severity (Fig. 1). Incidence of tail lesions on arrival to the second grower stage and on arrival to the finisher stage was 11.9% and 10.1%, respectively. No associations were observed between body weight or sex and the relative risk of developing a tail lesion (*P* > 0.05). The majority of tail lesions were mild in all production stages; 2.4% of pigs had mild tail lesions on transfer to the first grower stage while 10.9% and 8.5% of pigs had mild tail lesions on transfer to the second grower and finisher stage, respectively. No pig presented tail lesions on transfer to all three production stages (Fig. 1). Approximately, 0.06% of pigs presented tail lesions on transfer to both the first grower and finisher stage, 0.6% of pigs presented tail lesions on transfer to both the first grower and second grower stage, and 1.6% of pigs presented tail lesions on transfer to both the second grower and finisher stage (Fig. 1). The percentage of pigs that only presented tail lesions once was 1.9% on transfer to the first grower stage, 10.5% on transfer to the second grower, and 10.0% on transfer to the finisher stage.

### Patterns of ear and tail lesion severity across production stages

**Fig. 3:**
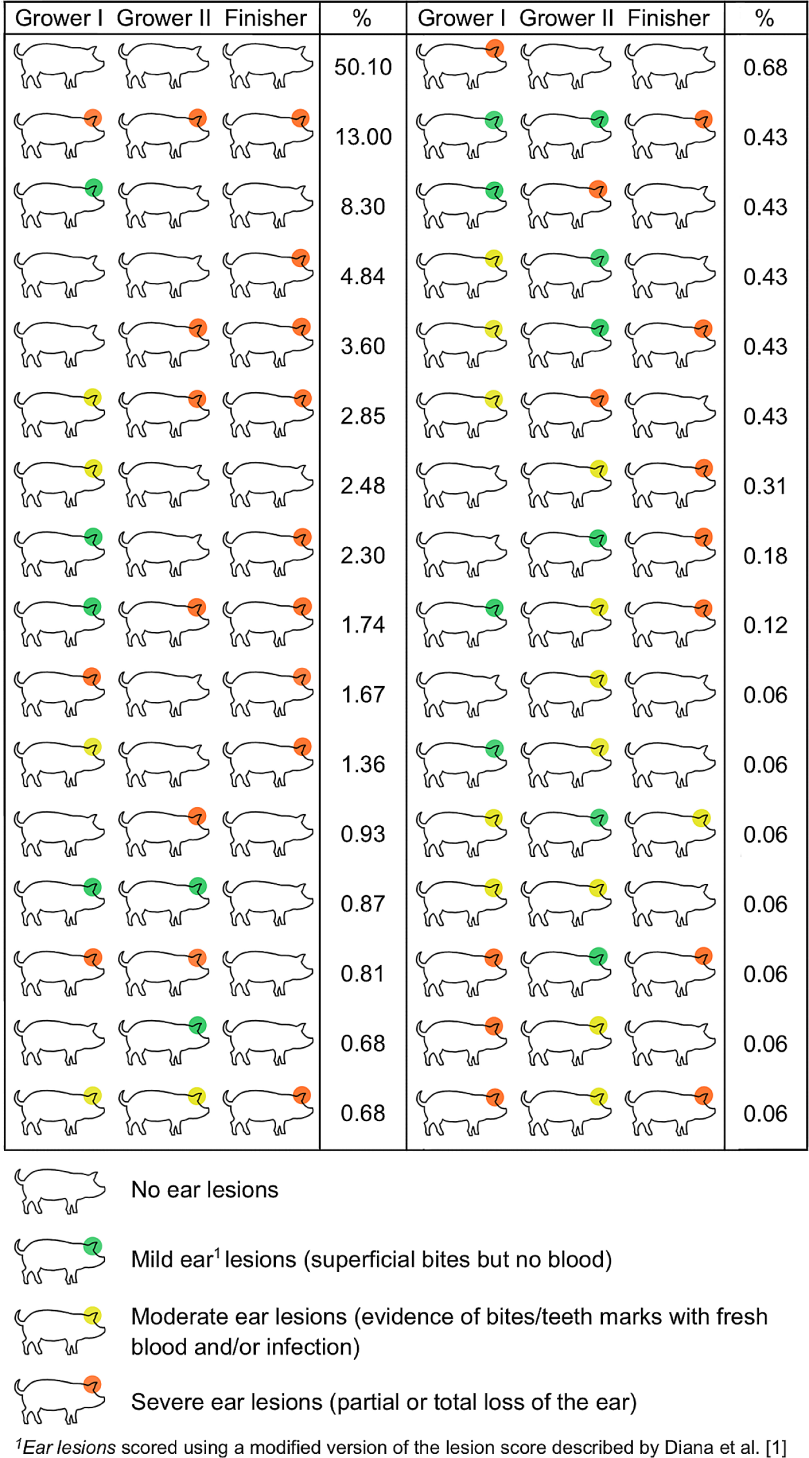
Different ear lesion score combinations observed in 1,612 grower-finisher pigs on transfer to the first grower (Grower I, 12 weeks of age), second grower (Grower II, 14 week of age), and finisher (Finisher, 18 weeks of age) stages on a commercial Irish pig farm

**Fig. 4:**
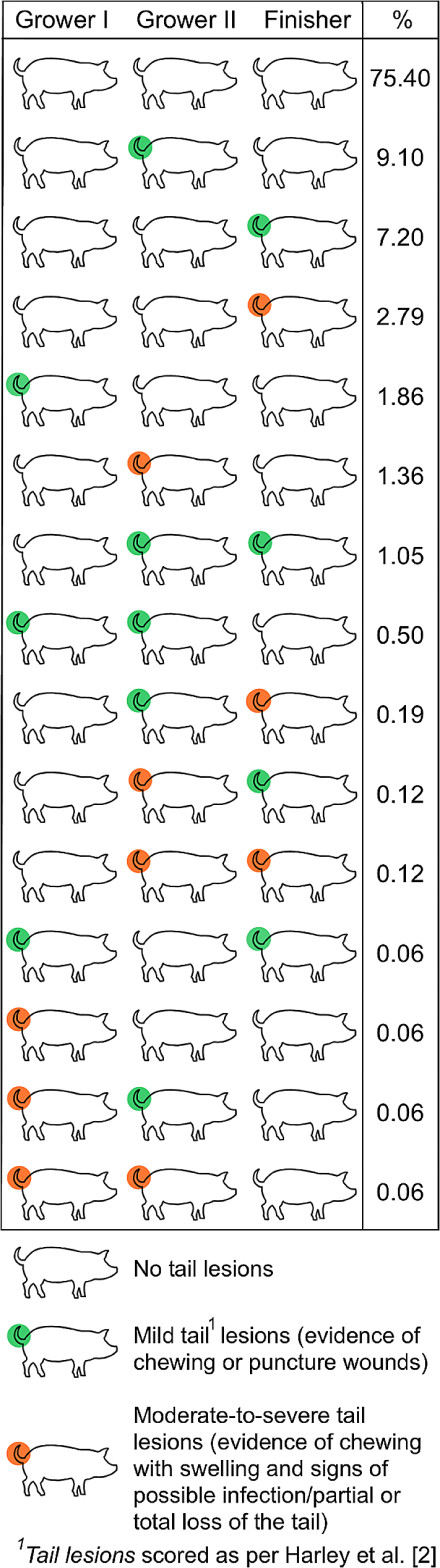
Different tail lesion score combinations observed in 1,614 grower-finisher pigs on transfer to the first grower (Grower I, 12 weeks of age), second grower (Grower II, 14 week of age) and finisher (Finisher, 18 weeks of age) stages on a commercial Irish pig farm

When considering the specific severity categories, we observed 32 different ear lesion score combinations (Fig. 3) and 15 different tail lesion score combinations across the three inspections (Fig. 4). In total, 50.1% of pigs did not present any ear lesions and 75.4% of pigs did not present any tail lesions at any of the inspections. The most frequently observed patterns of ear lesions were (i) pigs with severe ear lesions on transfer to the first grower, second grower and finisher stages (13% of pigs), (ii) pigs with mild ear lesions on transfer to the first grower stage and no ear lesions on transfer to the second grower or finisher stages (8.3%), and (iii) pigs with no ear lesions on transfer to the first grower or second grower stages but with severe lesions on transfer to the finisher stage (4.8%). The most frequently observed patterns of tail lesions were (i) pigs with no tail lesions on transfer to the first grower stage, mild lesions on transfer to the second grower stage and no lesions on transfer to the finisher stage (9.1% of pigs), (ii) pigs with no tail lesions on transfer to the first grower or second grower stages but with mild tail lesions on transfer to the finisher stage (7.3%), and (iii) pigs with no tail lesions on transfer to the first grower or second grower stages but with severe tail lesions on transfer to the finisher stage (2.9%).

## Discussion and conclusions

This case study provides a detailed account of the prevalence and patterns of ear and tail lesion development in pigs from 12 to 18 weeks of age on a single commercial grower-finisher farm in Ireland. While tail lesions were a major focus of pig welfare research in recent years [17], there is currently growing attention on ear lesions [3, 6, 7]. Previous studies reported a higher frequency of ear biting or ear lesions (depending on production stage) compared to tail biting or tail lesions in general [1, 18]. The prevalence of ear lesions observed in the current case study on transfer to the first grower (39.3%), second grower (28.6%) and finisher (33.6%) stages was also consistently higher than the prevalence of tail lesions (first grower: 2.6%, second grower: 12.5%, finisher: 11.7%). These figures reflect the prevalence irrespective of severity but it should be noted that lesions were mainly of the milder variety. This was particularly true for tail lesions, with only a low prevalence of moderate-to-severe tail lesions across all production stages. The severity of ear lesions generally increased as pigs progressed through the production stages. To put the observed prevalence into context, we can compare these results against other studies conducted in Ireland. There was a similar prevalence in a cross-sectional study of 31 farms in Ireland by van Staaveren et al. [1] for tail lesions, though ear lesion prevalence averaged below 10% in that particular study. This could be explained by the fact that van Staaveren et al. [1] inspected pigs for lesions from outside the home pen, while all animals were removed from their home pen and inspected individually in the current study. The prevalence of ear lesions (approx. 30–40%) is similar to that shown by Diana et al. [6] who used similar methods as in the current study, as well as more recent reports by Markland et al. (*personal communication, L. Markland)*. These more detailed measurements may allow for a more accurate assessment of the prevalence within a herd. Frequent ‘from outside the pen’ scans may be used by producers as an initial assessment to determine which pens require closer assessment. Self-assessment by producers if often recommended to continuously improve welfare on pig farms [19]. Additionally, increasing efforts are underway to investigate the potential of digital tools for automated assessment as a supporting aid [20–22], though this needs further development to be applicable on commercial farm conditions and comes with its own risks [23].

Previous accounts from cross-sectional studies [1, 3] and one longitudinal study [6] suggested that ear lesions are more common in the weaner/grower period, while tail lesions become the main problem in the finisher period. The same pattern is observed in the current study as also highlighted by the non-linear inverse relationship between body weight and risk of developing an ear lesion. For ear lesions there was a clear transition where lesions were initially mild to moderate in the first grower stage, but on transfer to the second grower and finisher stage ear lesions were mainly severe. Moreover, the detailed account of lesion progression throughout the different production stages revealed 32 possible combinations for ear lesions and 15 possible combinations for tail lesions. The longitudinal approach including inspections of individual pigs in the current study allowed us to look more closely at measures of incidence of ear and tail lesions which ranged between approx. 6–12%. For example, we were able to determine whether it was the same pigs that had lesions throughout the different stages, if new lesions had formed or lesions healed over time, or if mild lesions in earlier stages became more severe. The generally higher prevalence of ear lesions compared to tail lesions was also reflected in the finding that half of the pigs did not present any ear lesions on transfer to any of the production stages (i.e., consistently no lesions), while this was the case for approx. 75% of the pigs in regards to tail lesions. Moreover, 19% of pigs had ear lesions on transfer to each production stage (with the combination with the highest prevalence being severe ear lesions at each transfer), while no pigs were observed with tail lesions on transfer to each production stage. In general, pigs presented with ear lesions (3–5%) or tail lesions (0.1–1.5%) less frequently at two separate inspections depending on the transfer combination (e.g., transfer to both first and second stage grower, second stage grower and finisher, first stage grower and finisher). Finally, there was a low prevalence of pigs that were affected by both tail and ear lesions on transfer to first grower (0.8%), second grower (3.4%) and finisher (4.0%) stages. The higher co-occurrence in later stages likely reflects the age-dependent increase in tail lesions in particular. Previous studies suggested a link between tail and ear biting [24, 25] though this is not always the case for tail and ear lesions [26], and such studies frequently assessed lesions at a pen level. The low prevalence of co-occurrence in the current study suggests that the aetiology differs between the two behaviours, heterogeneity within both tail and ear biting behaviour [21, 27], and that there is not necessarily a ‘victim profile’ that makes certain pigs more likely to receive both type of lesions. It would have been interesting to assess the profiles of the ‘biter’ pigs as well, similarly as Ursinus et al. [9] did for tail biting alone, but this was outside of the scope of the current study.

For ear lesions it is important to note that these lesions may intensify quite quickly and persist throughout the production stages. Pigs with mild ear lesion on transfer to the first grower stage could have severe ear lesions at transfer to the second grower stage 2 weeks later. The initial damage, likely caused by ear biting, may lead to opportunistic bacteria populating the area, disruption blood supply to the ear and impairing the immune system to the point that necrosis establishes a few weeks later [5, 28]. This may explain the associations observed between ear lesions and ear necrosis [7, 28, 29]. Lesions caused by behavioural activity (i.e. damaging ear biting) may thus deteriorate quickly and require early intervention. Producers may be more aware of tail biting and have more strategies to address the problem [3, 30, 31]. In contrast, the issue of ear biting has not received much attention and there are no management strategies to address this behaviour [21, 32].

In certain cases, mild ear and tail lesions were recorded at one inspection but not at the subsequent one(s). For cases where mild lesions were observed on transfer to the first grower stage but not at subsequent transfer to the second grower and finisher stages, it would be logical to assume that the lesions healed over time. However, in cases where lesions reappeared at inspection on transfer to the finisher stage, it is likely that the lesions were merely missed (due to e.g., dirt, movement) at inspection on transfer to the second grower stage. Another possibility is that the mild lesions healed (and hence were not observed on transfer to the second grower stage), but new lesions formed between the inspection on transfer to the second grower and finisher stage. Similarly, Ursinus et al. [9] reported that pigs with tail lesions remained relatively consistent from weaning to slaughter, but some pigs were no longer victims at later stages. It should be acknowledged that there were also some cases, albeit at a much lower frequency, where a pig had severe lesions at one inspection, but no lesions at the next inspection. This could be because we combined severe scores which were less frequently observed. As such, for tail lesions the moderate-to-severe category included cases with signs of chewing, swelling and/or infections where healing may still have been possible. Additionally, only the presence of fresh lesions was recorded. This could explain why pigs with partial ear loss were recorded as having no lesions in a subsequent inspection, if lesions were healed with skin intact (no fresh blood). It could be argued that these healed lesions should still be considered severe, however, in the current study we chose to focus on fresh lesions.

In summary, this longitudinal study highlighted that ear lesions were more of a problem than tail lesions throughout production on the particular commercial farm under investigation. Identification of mild lesions by frequent inspection may aid in earlier intervention and application of management strategies. The high number of observed patterns of ear and tail lesions suggests large individual variability in lesion progression that may be more complex than originally thought. It should be noted that results of this case study cannot be generalized as it was performed on a single grower-finisher farm. While this farm was typical for the grower-finisher systems in Ireland, it should be acknowledged that integrated sow-to-finish farms are more common in Ireland (approx. 200 integrated farms and approx. 80 finisher farms in 2020) [33]. However, these systems are also found in other countries and consist of similar indoor group housing with fully-slatted floors as is common in Europe [34]. As such, while patterns of lesions may differ from farm to farm, increased awareness of the complex and dynamic lesion progression may still be of interest to those involved in improving pig welfare. Further research where pigs are followed longitudinally from birth in regard to tail and ear lesions may give more insights into lesion development, considering that approx. 40% of pigs already had some sort of lesion (ear or tail) upon arrival at the grower-finisher facility. While not possible in the current observational study, it would also be interesting to assess the association between the percentage of pigs in a pen with lesions and the likelihood of new pigs in the pen developing lesions, as it is suggested that lesions may cause tail or ear biting behaviour to spread [4]. Additionally, pigs were tail docked in the current study and with increasing focus on rearing non-docked pigs it is expected that results would differ if pigs were reared with intact tails. Understanding lesion development in non-docked pigs may be even more crucial to be able to intervene earlier.

## Data Availability

The datasets used for the results presented in this case study are available from the corresponding author upon reasonable request.
